# Virtual rehabilitation for patients with osteoporosis or other musculoskeletal disorders: a systematic review

**DOI:** 10.1007/s10055-024-00980-7

**Published:** 2024-04-07

**Authors:** Eléa Thuilier, John Carey, Mary Dempsey, John Dingliana, Bryan Whelan, Attracta Brennan

**Affiliations:** 1 School of Computer Science, University of Galway, Galway, Ireland; 2 School of Medicine, University of Galway, Galway, Ireland; 3 School of Engineering, University of Galway, Galway, Ireland; 4 School of Computer Science and Statistics, Trinity College Dublin, Dublin, Ireland

**Keywords:** Virtual rehabilitation for older adults, Osteoporosis, Musculoskeletal disorders, Patient engagement, Exergames, VR/AR

## Abstract

This study aims to identify effective ways to design virtual rehabilitation to obtain physical improvement (e.g. balance and gait) and support engagement (i.e. motivation) for people with osteoporosis or other musculoskeletal disorders. Osteoporosis is a systemic skeletal disorder and is among the most prevalent diseases globally, affecting 0.5 billion adults. Despite the fact that the number of people with osteoporosis is similar to, or greater than those diagnosed with cardiovascular disease and dementia, osteoporosis does not receive the same recognition. Worldwide, osteoporosis causes 8.9 million fractures annually; it is associated with substantial pain, suffering, disability and increased mortality. The importance of physical therapy as a rehabilitation strategy to avoid osteoporosis fracture cannot be over-emphasised. However, the main rehabilitation challenges relate to engagement and participation. The use of virtual rehabilitation to address such challenges in the delivery of physical improvement is gaining in popularity. As there currently is a paucity of literature applying virtual rehabilitation to patients with osteoporosis, the authors broadened the search parameters to include articles relating to the virtual rehabilitation of other skeletal disorders (e.g. Ankylosing spondylitis, spinal cord injury, motor rehabilitation, etc.). This systematic review initially identified 130 titles, from which 23 articles (involving 539 participants) met all eligibility and selection criteria. Four groups of devices supporting virtual rehabilitation were identified: a head-mounted display, a balance board, a camera and more specific devices. Each device supported physical improvement (i.e. balance, muscle strength and gait) post-training. This review has shown that: (a) each device allowed improvement with different degrees of immersion, (b) the technology choice is dependent on the care need and (c) virtual rehabilitation can be equivalent to and enhance conventional therapy and potentially increase the patient’s engagement with physical therapy.

## Introduction

As one of the most common skeletal disorders in the world (Ho et al. 2021), osteoporosis is a disorder affecting the body’s musculoskeletal (muscular and skeletal) system (Ho et al. 2021) and results in the inner structure of bones breaking down, becoming more fragile and prone to breaking (Sözen 2017; Erjiang et al. 2020; Kanis et al. 2021). Osteoporosis is called a silent disease as there is usually no symptom until an osteoporotic fracture occurs (Sözen 2017; Erjiang et al. 2020). An osteoporotic fracture, also known as fragility fracture, usually occurs after a fall (Sözen 2017; Erjiang et al. 2020). While falls can occur at any time in life, their severity and frequency increase with ageing (Australian Commission on Safety and Quality in Health Care 2009). A fall is defined in this context as any event resulting in a person coming inadvertently to rest on the ground or any other lower level (Sözen 2017; Erjiang et al. 2020; Australian Commission on Safety and Quality in Health Care 2009). When the fall is the cause of the fracture, it is called an ‘injurious fall’ (Australian Commission on Safety and Quality in Health Care 2009). In 2019, 25.5 million women and 6.6 million men were estimated to have osteoporosis (European Union plus Switzerland, plus United Kingdom), whilst 4.3 million patients suffered from an osteoporotic fracture (Willers et al. 2022). The hospitalization rate for osteoporotic fractures is predicted to increase by 150% in 2046 (McCabe et al. 2020). Meanwhile, the projected growth in the population up to 2034, for those aged over 50 years, is 11.4% for men and women (Willers et al. 2022). Physical exercise is a powerful non-pharmaceutical fracture prevention strategy for people with osteoporosis or those at risk of falls (Dionyssiotis et al. 2014). However, despite the importance of physical exercise, the participation in and adherence to an exercise regimen by older adults is often low due to; a lack of time and motivation, boredom, a fear of falling, and/or environmental considerations such as inconvenience, accessibility, safety and/or cost (Valenzuela et al. 2018). To address this lack of participation and adherence, virtual rehabilitation is gaining in popularity (Valenzuela et al. 2018; Staiano and Flynn 2014). 

Virtual reality (VR) is defined as an application that allows users to navigate through and interact with a completely virtual environment (Baus and Bouchard 2014). Meanwhile, augmented reality (AR) is described as a system which enhances the physical environment by overlaying virtual artefacts on the user’s perception of the real world (Baus and Bouchard 2014). Both VR and AR use virtual environments (VEs) which are accessible through immersive devices (Baus and Bouchard 2014) and can enable different forms of interaction through the capture of body pose and/or gesture (Baus and Bouchard 2014; Jerald 2015). A head-mounted display (HMD) (e.g., Oculus, Hololens, etc.) can be used to engage the user with a fully immersive audio-visual experience through movement detection (i.e. sensors, camera) and real-time visual, audio (and sometimes haptic) feedback (Jerald 2015). Less immersive experiences can be provided through the use of controllers (i.e. gaming controllers, Kinect camera, Wii balance board) and a screen (e.g. computer monitor, television screen, projection, etc.) displaying the environment in front of the user (Jerald 2015). Many interaction controllers can be used for the various levels of immersion including wearable inertial measurement unit (IMU) sensors [e.g. Xsens (Roetenberg et al. 2009)], body tracking cameras (e.g. Intel RealSense, Leap Motion, Kinect (Han et al. 2013)), and human pose detection libraries [e.g. OpenPose (Losilla and Rosique 2019)].

Virtual rehabilitation has been defined as the ability of virtual reality (VR) to provide therapy to patients using its hardware and simulation (Mubin et al. 2019). Exergames are games delivered through AR, VR or other forms of videogames and require the participants to be physically active (Song et al. 2011). Virtual rehabilitation and exergames allow various exercises mixing games and therapy, by enabling control (i.e., adaptation to the patients’ needs) and contact with the environment (i.e., real-time feedback) (Mubin et al. 2019). The potential of exergames for rehabilitation has been studied for older people from the viewpoint of physical and mental improvement (Staiano and Flynn 2014; Song et al. 2011).

To the best of our knowledge, there is no systematic review which explores and analyses existing technology-based approaches and exercises using virtual rehabilitation to support the physical rehabilitation of older adults and post-menopausal women with an emphasis on osteoporosis. As osteoporosis is a systemic skeletal disorder, this review addresses the following question; What is the most effective way to design virtual rehabilitation to obtain physical improvement (e.g. balance and gait) and support engagement (i.e. motivation) for people with musculoskeletal disorders?

## Methodology

In this study, the authors followed the Preferred Reporting Item for Systematic Reviews and Meta-Analyses (PRISMA) guidelines (Page et al. 2021). The PICO (patient/population, intervention, comparison, and outcomes) methodology was also used to define the research question. The term ‘older adults’ refers to people over 65 years old, and/or a vulnerable population when it comes to pharmacotherapy (Singh and Bajorek 2014).

### Databases search

**Fig. 1:**
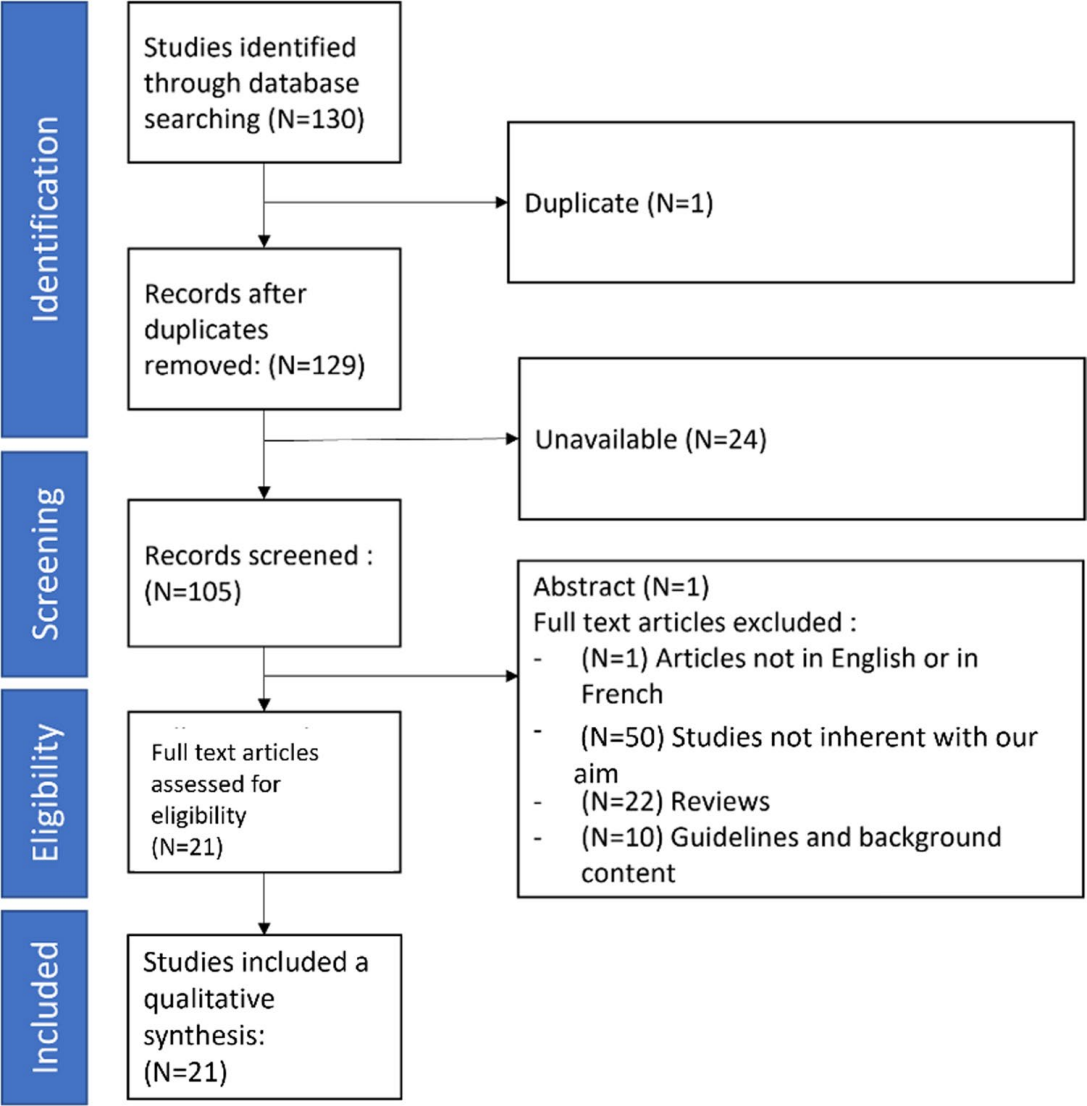
Flow chart of study's selection following PRISMA guidelines (Page et al. 2021)

Given the clinical underpinnings of this review, we particularly focussed on the PubMed database, while also searching Google Scholar, and ACM. These three databases were searched between September and December 2021 (ET) and then validated by (AB, JJC, JD and MD) (Fig. 1). The following keywords and their combination were used: ‘Rehabilitation’, ‘AR’, ‘VR’, ‘Osteoporosis’, ‘Ageing’, ‘Therapy’, ‘Exercises’, ‘Fall Prevention’, ‘Human–Computer Interaction (HCI)’, ‘Task-oriented’, ‘Ankylosing spondylitis’, ‘Spinal Cord Injury’, ‘Balance’, ‘Muscular and Skeletal (MSK)’ and ‘Motion capture’. The inclusion criteria comprised: (a) content that focussed on physical exercise rehabilitation (e.g., balance, gait, muscle strength, flexibility); (b) VR/AR or exergames for therapy; (c) applicable to an older population; (d) accessible in open source; and (e) articles written in English or French. The exclusion criteria were: (a) studies not affecting physical rehabilitation; (b) content that focuses only on technology and not on therapy and (c) review or guideline content.

All selected articles were published between 1986 and 2021. This period was selected as researchers started to explore the use of VR in non-entertainment settings (e.g. psychiatric treatment (Maples-Keller et al. 2017) in 1986.

Note: As there was a paucity of literature applying virtual rehabilitation to patients with osteoporosis, the authors broadened the search parameters to include articles relating to the virtual rehabilitation of other skeletal disorders (e.g., Ankylosing spondylitis, spinal cord injury, motor rehabilitation, etc.). These disorders were validated by this paper’s authors having a clinical background (JJC, BW) and are comparable to the physical therapy needs of patients with osteoporosis.

The selection process comprised (Fig. 1);

(i) Identification: 130 articles (60% from PubMed, 26% from Google Scholar and 14% from ACM) were selected based on the keyword search and the inclusion and exclusion criteria. However, one article was excluded due to duplication reasons.
(ii) Screening: In this stage, 24 articles were excluded due to access issues (i.e., not open-access and inaccessible by the authors’ institution).
(iii) Eligibility: Of the remaining 105 (55% from PubMed, 28% from Google Scholar and 17% from ACM) articles, 59 were excluded based on details of the methods, studies and results; 25 were considered incompatible with this study for not focusing on the training or the design of the therapy or without any results, 22 were review articles, 10 were guidelines which focused on osteoporosis, 1 was rejected based on language issues, and 1 was excluded because it presented only an abstract (no full article). The remaining 46 articles were reviewed once more. 25 articles were excluded because they did not focus on physical training and older adults. This resulted in 21 articles (83% from PubMed, 13% from Google Scholar and 4% from ACM) being selected for this review.

## Results

The 21 articles screened and included in this review cover a population total of 526 patients, aged between 18 and 88 years, 15 of the studies were exclusively related to older people and post-menopausal women while 7 focused on a mixture of younger and older populations. Note: The definition of older adults in this review is 65 years old and over (Singh and Bajorek 2014).

As outlined in the inclusion criteria, the selected articles do not focus exclusively on patients with osteoporosis but are extended to other care needs which also require physical rehabilitation, and/or training exercises. Hence the inclusion of eight different care needs in this study; fall prevention, general motor rehabilitation, spinal cord injury, post-stroke, shoulder abduction, ankylosing spondylitis, orthopaedic rehabilitation post-total knee arthroplasty (TKA) and Parkinson’s disease. Fall prevention and general motor rehabilitation are part of the useful training exercises for osteoporosis as they improve balance, gait, and/or motor abilities. Moreover, physical therapy for ankylosing spondylitis, spinal cord injury, post-stroke, shoulder abduction and orthopaedic rehabilitation post-total knee arthroplasty (TKA), is similar to that required for osteoporosis.

The studies included in this review have been categorised into the following groups; studies using a head-mounted display for virtual rehabilitation (see §3.1); studies using a Balance board (such as a Nintendo Wii Fit Plus) (see §3.2); studies using a camera for body recognition (see § 3.3) and studies using other examples of devices (e.g. data gloves) (see §3.4). In each group, the studies will be presented with the population, the setup, the design of the experiment and outcomes and results.

To avoid unnecessary duplication, we first define the measurement approaches prior to presenting them in the associated study results’ tables.

### Group 1. use of a head-mounted display (HMD)

A summary of the study population and setup for Group 1 is shown in Table 1.

**Table 1:** Summary of the population and setup for Group 1 (HMD)

Study	Sample size	Age profile	Care need	Head mounted display
Blomqvist et al. (2021)	8	66–88 years old	Fall prevention	Microsoft Hololens—mixed reality headset
Yoo et al. (2013)	21	70 + years old	Fall prevention	I-Visor FX601—video glasses
Jung et al. (2012)	25	52–68 years old	Post-stroke	Accupix MyBud—video glasses
Lee et al. (2017)	30	70–81 years old	Fall Prevention	I-Visor FX601—video glasses

A summary of the interventions for each study in Group 1 is shown in the Table 2.

**Table 2:** Summary of the intervention for Group 1 (HMD)

#Sessions/ #Week /Duration (min)	Experimental group			Control group		
	Intervention	Focus	Detail	Intervention	Focus	Details
Blomqvist et al. (2021)						
12/6/20	Exercise 1: ‘catch’ a virtual ball with the laser Exercise 2: Burst a moving ball by pointing at it with a virtual laser	Balance	Exercise 1: Sideways motion of upper body Moving CoG in a fixed position Exercise 2: Rotation of head and upper body	No control group		
Yoo et al. (2013)						
12/12/60	Patients repeat the AR-based Otago exercises displayed by the device	Muscle Strength and Balance	Knee extension for front knee strengthening, knee flexion for back knee strengthening, hip joint, Abduction for side hip strengthening, Plantar flexion for calf raises of the ankle, Toe raises dorsiflexion of the ankle Walking exercises (backward, turning around, heel-to-toe, heel, toe, heel-to-toe backward, stair), sit to stand and one leg stand	Otago exercise	Muscle Strength and Balance	Knee extension for front knee strengthening, knee flexion for back knee strengthening, hip joint, Abduction for side hip strengthening, Plantar flexion for calf raises of the ankle, Toe raises dorsiflexion of the ankle Walking exercises (backward, turning around, heel-to-toe, heel, toe, heel-to-toe backward, stair), sit to stand and one leg stand
Jung et al. (2012)						
15/3/30	Virtual reality treadmill training	Balance, balance self-efficacy	Patients watch the virtual reality program displaying a park stroll and wall on the treadmill	Treadmill training	Balance, balance self-efficacy	
Lee et al. (2017)						
12/12/60	Patients practice a AR-based Otago exercises displayed on the device	Muscle strength	Knee flexion exercises Walking exercises (backward, turning around, heel-to-toe, heel, toe, heel-to-toe backward, stair), and sit to stand	Self-Otago exercise	Muscle strength	Knee flexion exercises Walking exercises (backward, turning around, heel-to-toe, heel, toe, heel-to-toe backward, stair), and sit to stand
				Patients practice a yoga program	Balance, muscle strength	Cat pose, tree pose, cobra pose, triangle pose, twist pose, leg stretch, and pigeon pose

One study (Blomqvist et al. 2021) decided to keep the patients in one group (i.e. no control group). As the patients interacted with the virtual environment, they were required to track and catch an object. The training sessions had increasing levels of difficulty. Two studies (Yoo et al. 2013; Jung et al. 2012) separated the patients into two study groups. Yoo et al. (2013) developed Otago exercises using AR, while Jung et al. (2012) employed a VR treadmill exercise. Meanwhile, the control groups for both studies performed similar exercises to the intervention groups using conventional methods. The Otago exercise programme was developed by the New Zealand fall prevention research group to reduce falls in older adults (over 65 years of age) (Gardner et al. 2001). It consists of 17 strength and balance exercises and a walking progressive-resistance program (Yoo et al. 2013; Lee et al. 2017; Gardner et al. 2001). This program also includes five strengthening exercises (knee extensor, knee flexor, hip adductor, ankle plantar-flexors (calf raises) and ankle dorsiflexors (toe raises) and 12 balance exercises (knee bend, backwards walking, walking, and turning around, sideway walking, tandem stance (heel-toe stand), tandem walk (heel-toe walk), one leg stand, heel walking, toe walk, heel-toe walking backwards, sit to stand, stair walking) described in Gardner et al. (2001). The AR-Otago exercises used in Yoo et al. (2013) focussed on muscle strengthening and balance training. The VR treadmill exercise in Jung et al. (2012) comprised a conventional treadmill training without control over the slope for the control group and a walk-in-a-park stroll for the intervention group. Lee et al. (2017) separated their patients into three groups: one intervention group (AR-Otago) and two control groups (Yoga exercises and self-Otago). The AR-based group (with technology) and the self-Otago group practiced exercises focussing on knee flexion, walking in different conditions, and standing up. Yoga exercises were also proposed for the third group i.e. seven distinct positions proposed without the use of technology.

The four studies in this group used a combination of the following measurement approaches:

- Falls Efficacy Scale-International (FES-I): is a 16-items scale used to measure the confidence of the patient in performing daily activities without falling (Delbaere et al. 2010). An FES-I score ranges between 16 and 64 (where a high score indicates low confidence and a high risk of falling) (Delbaere et al. 2010).
- Falls Efficacy Scale Swedish Version (FES-SV): is a slight variation of the FES-I, adapted for the Swedish language.
- Morse Fall Scale (MFS): is a scale to assess a patient's likelihood of falling based on their medical history (i.e. their history of falling, previous diagnoses, ambulatory aid needs, gait state and mental state) (Morse et al. 1989). An MFS score ranges between 0 and 125. The patient is considered at minimal risk for any score under 25, at moderate risk when the score is between 25 and 45, and at high risk of falling when the MFS score is greater than 45 (Morse et al. 1989).
- Activity-Specific Balance Confidence (ABC): is a 16-item structured questionnaire used to measure an individual's confidence in performing daily activities without losing balance (Wildschut et al. 2020). The scores range from 0 to 100%. The patient is considered to have a high level of physical functioning for any score over 80%, a moderate physical functioning when the score is between 50 and 80%, and a low level of physical functioning when the ABC score is under 50%.
- Force Platform: first measures the force exerted by the body on the ground and then determines the centre of pressure, the centre of gravity and the balance (Browne and O’Hare 2000). Force Plate and Ergopower Platform are similar in functionality to a Force Platform.
- Berg Balance Scale (BBS): is a 14-item scale used to objectively determine the patient's ability to safely balance during a series of pre-determined tasks (Berg et al. 1989). The patient is considered at great risk of falling when the BBS score is under 45; they are considered to have a functional balance when the BBS score is greater than 56.
- Short Physical Performance Battery (SPPB): is a series of time-recorded exercises [such as sit and stand and balance testing with the feet in a certain position (i.e. feet together, semi-tandem, full-tandem)] used to evaluate lower extremity function and mobility in older people (Gómez et al. 2013).
- Time Up and Go (TUG): is a test to determine fall risk and measure a person’s balance. The TUG test consists of a sit-to-stand and walking exercise. An older adult who takes more than 12 seconds to complete the TUG test is considered at risk of falling (Kear et al. 2017)
- Gait Rite system: is a three meters walkway used to measure spatio-temporal parameters (i.e. gait, velocity, cadence, step length and stride length) (Yoo et al. 2013; McDonough et al. 2001).
- Raptor 4S (optical camera): detects and analyses body movements (Topley and Richards 2020).
- Manual muscle test: is an important evaluating tool to assess impairments and deficits (including strength, power, and endurance) in muscle performance (Robertson 1984). By working against gravity or against an opposing manual resistance, the Manual Musce test evaluates the patient’s available range of motion. A score of 5 indicates that the patient’s muscle performance is considered to be of normal strength while a score of 0 indicates no strength (Robertson 1984).
- Maximum voluntary contraction test: is a measures muscle strength using EMG electrode data (Boettcher et al 2008).

The feedback from the patients and physiotherapists regarding the interventions is presented in Table 3.

**Table 3:** Summary of measurements and results for Group 1 (HMD)

Study	Measurement	What was measured	Results
Blomqvist et al. (2021)	Interviews	Patients’ opinions/comments on practicality around balance training	Six patients thought that 15–20 min was adequate, not too long
			One patient said it could have been longer
			Five patients said that training twice a week at a fixed time was good and easy to plan
			Two patients felt stuck with the fixed scheduled training and indicated that they would prefer more frequent and shorter training sessions
		Physiotherapists’ opinions/comments on practicality around balance training	A more frequent and shorter training program would mitigate against patients getting tired, experiencing pain and/or losing concentration
		Patients’ opinion/comments on the experience of the two training games: exercise 1	Four patients had difficulty moving the ball from the centre to the side
			All patients felt that the exercises went well in the beginning when the ball did not move much to the side. The patients also reported that the exercises soon became predictable (balls alternatively coming from left and right), repetitious and boring;
			Five patients felt that the ball disappeared too easily from the screen
		Physiotherapists’ opinion/comments on the experience of the two training games: exercise 1	The training program was difficult for motor performance
			Once the exercises became too static, motivation was decreased
		Patients’ opinion/comments on the experience of the two training games: exercise 2	All patients expressed that they had to concentrate in order to follow the trajectory of the ball. They also reported no difficulty when standing still but the training became more difficult while walking or standing on one foot
			Five patients felt that the training was more positive and interesting while walking than standing still. They reported that the walking exercise was fun while the standing still exercise was easy but boring
		Physiotherapists’ opinion/comments on the experience of the two training games: exercise 2	The training program was beneficial to the patients. An increase in the choice of exercises, in addition to increased difficulty would potentially increase motivation and concentration. For these games to be effective, the patients would need to understand that they have to challenge themselves and have clear goals
		Patients’ opinions/comments on the increasing level of difficulty of balance training	Every patient experienced a gradual increase in the level of difficulty. However, none of the patients fully managed the training on one leg
		Patients’/opinions/comments on the feedback given during the training	Five patients felt that the feedback given during the training was positive but could be a lot more clear regarding what was good/bad
			The opinion about the audio feedback was mixed, with some feeling that it did not match with the visuals while others thought it was appropriate
		Patients’ opinion/comments on the experience of the physical and technical aspect of the Hololens and its application	Six patients felt that wearing the Hololens was not a problem although the display was heavy to wear
			Every patient said that the Hololens was a poor fit. The hand and fingers command were difficult to execute
			Two patients suffered pain through wearing the Hololens; one patient suffered pain in the nasal root, whilst the other patient suffered forehead pain
			Three patients reported the need for outside help (e.g. feedback from a therapist, help with the device) during the training
			The Hololens took time to calibrate and was sensitive to any change. The patients found it frustrating to have to re-calibrate the HMD during the training
		Physiotherapists’ opinion/comments on the experience of the physical and technical aspect of the HoloLens and its application	The pain from the Hololens could be avoided by using padding for the nose or helmet
			The main weaknesses of the Hololens included: the small screen, a small field of view and inadequate contrast
		Patients’/Physiotherapists’ opinion/comments on clarity	Several patients asked for greater clarity in the instructions regarding how to start, what to do, what the scores meant and when you had successfully completed the training
		Patients’ opinion/comments on exercising at home	Six patients considered practicing balance exercise at home with the Hololens after the study and would recommend it to their friends
			Two patients explained that the technology was not an issue, but the motivation and the fear of losing the social part of the training could be a problem
			Some patients felt that the technology could be useful if someone wanted to follow up the training
		Physiotherapists’ opinion/comments on exercising at home	The technology is helpful if the instructions are clear (with video and sound) and the training difficulty increases as appropriate. The HoloLens is easy, convenient, safe, cost-effective and fun
		Patients’ opinion/comments on usability of new technology	All patients considered the training instructions to be well worded. However, the hand and finger movements tended to be difficult, in addition to manoeuvring in the menus
			Six patients felt that they would be able to use the Hololens alone at home
	BBS	Balance	Two patients increased their score
	SPBB-SV	Balance	Two patients improved their score whilst four patients stabilised and/or kept good score
	FES-SV	Falls	Five patients improved their score
	Ergopower platform	Balance	Semi-standing with their feet: four patients decreased the sway (in mm) between before and after the training and two patients increased it
			Tandem standing with their feet: four patients decreased the sway (in mm) between before and after the training and one patient increased it
Yoo et al. (2013)	BBS	Coordinate data on major upper body landmark (used to measure the shoulder joint flexion)	Improvement in the BBS score in both groups, but greater improvement in the experimental group
	Gait Rite System	Gait, velocity, cadence, step length and stride length	Improvement of velocity, cadence, step length and stride length in both groups
	FES-I	Falls	Improvement of fall risk in both groups but greater in the experimental group
Jung et al. (2012)	TUG	Balance	Significant increased improvement in balance. Higher improvement in the experimental group
	ABC	Balance self-efficacy	Significant increased improvement in balance self-efficacy. Higher improvement in the experimental group
Lee et al. (2017)	Force Plate	Balance	EO-COP significantly decreased in AR group and Yoga group
			EC-COP, EO-SD, EO-HoE decreased only in the AR group
	MFS	Falls	Meaningful improvement for the AR group
	MMT	Muscle strength	Knee flexion and ankle dorsiflexion strength significantly improved in the three groups

In order of appearance: BBS: Berg Balance Scale; FES-I: Fall Efficacy Scale; EMG: Electromyography; TUG: Time Up and Go; ABC: Activities-Specific Balance Confidence Scale; EO-COP: Eyes Opened-Centre of Pressure; EC-COP: Eyes Closed-Centre of Pressure; EO-SD: Eyes Opened Standard Distance; EO-HoE: Eyes Opened-Height of Ellipse; MFS: Morse Fall Scale; MMT: Manual Muscle Test

70% of the patients from Blomqvist et al. (2021) mentioned that the feedback provided during the training was positive and could help to motivate them to train more. However, the remainder of the patients felt that the feedback could be clearer and/or that the audio feedback did not always match the situation (Blomqvist et al. 2021). Each of the four studies concluded that the virtual therapy was not only feasible (Blomqvist et al. 2021) but provided opportunities to improve balance (Yoo et al. 2013), gait (Yoo et al. 2013), fall efficacy (Yoo et al. 2013), strength (Jung et al. 2012) and/or symmetry (Jung et al. 2012) while increasing engagement for the physical therapy with meaningful exercise (Blomqvist et al. 2021; Yoo et al. 2013; Jung et al. 2012; Lee et al. 2017).

### Group 2. use of a balance board

Balance boards such as the Nintendo Wii Fit plus are often used in exergames for patient rehabilitation (Yen et al. 2011; Wall et al. 2015; Van den Heuvel et al. 2014). Six selected articles utilized a balance board for their study (Table 4).

**Table 4:** Summary of the population and setup for Group 2 (Balance board)

Study	Sample size	Age profile	Care need	Setup
Lee et al. (2015)	24	33–62 years old	Post-Stroke	Balance board (Wii Fit Balance)
(Yen et al. (2011)	42	55–84 years old	Parkinson	Balance board
Szturm et al. (2011)	30	68–85 years old	Motor rehabilitation	Balance board
Cho et al. (2014)	32	70–74 years old	Motor rehabilitation	Balance board (Wii Fit Balance)
Wall et al. (2015)	5	50–64 years old	Spinal cord injury	Balance board (Wii Fit Balance)
Van den Heuvel et al. (2014)	33	59–77 years old	Parkinson	Balance board

All studies required a screen to display the training. The Wii balance board is a game controller for the Nintendo Wii system, released in 2007, and has been employed in clinical rehabilitation settings worldwide (Bartlett et al. 2014). The Wii balance board contains similar components to a typical force platform (Bartlett et al. 2014). A summary of the interventions for each study is shown in Table 5.

**Table 5:** Summary of the intervention for Group 2 (Balance board)

#Session/#Week /Duration (min)	Experimental group			Control group		
	Intervention	Focus	Details	Intervention	Focus	Details
Lee et al. (2015)						
30/ 6/ 90	The patients participate in a Wii fit plus program	Balance	Program includes sitting posture, knee bend and the leg knee extend, tightrope walking, penguin teeter-totter seesaw, balance skiing, rolling marble board, balance Wii	Warming up and stretching pre-exercise	Lower extremities exercising	Warm up/ cool down, range-of-motion, stretching exercise
	General Therapy	Movement therapy, synergic pattern of spastic muscle, motor control	Brunnstrom one on one movement therapy (Pandian et al. 2012)	General exercise therapy	Movement therapy, synergic pattern of spastic muscle, motor control, Motor function and stretching	Brunnstrom one on one movement therapy Neurodevelopmental therapy Proprioceptive neuromuscular facilitation
		Motor function and stretching	Neurodevelopmental therapy Proprioceptive neuromuscular facilitation	Task-oriented training	Balance	Sit-to-stand from different heights, task training in standing, balance training on an unstable surface, lifting a leg in place, kicking a ball, stair climbing and descending
Yen et al. (2011)						
6 / No information / 30	Stretching and warming up exercises	Flexibility of the trunk, thighs, and shanks, Stretching	Warming up exercise, stretching of trunk, thighs, and shanks	Stretching and warming up exercises	Flexibility of the trunk, thighs, and shanks, Stretching	Warming up exercise, stretching of trunk, thighs, and shanks
		Balance	"*Bang Bang Ball*”: Target one to five virtual balls on a virtual plate with a hole in the central position. The plate can be move by weight-shifting in different direction "*Simulated Board Driving games*”: Driving simulation in an outdoor simulated environment with straight and circular movement and multiple turns to control weight-shifting in daily environment	Conventional balance therapy	Balance, Weigh-shifting, Ankle strategy, Adaptability	Static stance; Dynamic weight shifting; external perturbation
Szturm et al. (2011)						
16 / 8/ 45	Dynamic balance training	Balance, weight shifting, postural stability, continuous automatic postural adjustments	"*Under pressure*" game: the patient is required to shift his/her weight to move a game sprite left or right on the display to catch an object falling from the top of the screen “*Memory match*” game: The patient shifts his/her weight in different directions in order to move the on-screen centre of pressure trajectory marker to a square to turn its card and display the picture and then move to a second square to find its match "*Balloon burst*" games: the patients move the COP in both AP and ML directions to intercept and burst the balloons	Conventional training	Balance, Muscle strength, endurance, gait	Hip flexion, side-left raises, squat, standing up from a chair and sitting down in the chair without using hands Thera-band, leg-weights Cycle ergometer Unsupervised walking program and gait re-education with parallel bar including heel-to-toe and lateral (crossover) walking, use of raised platforms for step up
Cho et al. (2014)						
24/8/30	Wii Fit training	Balance	Ski slalom, table tilt, balance bubble	No training		
Wall et al. (2015)						
14/7/60	Wii Fit training	Dynamic balance, Speed, Reaction Time, Endurance	Penguin plunge, Segway, Island bike, run, rolling down river, tightrope, obstacle course, ski jump, skiing, tilt table	No control group		
Van den Heuvel et al. (2014)						
10/10/60	Dynamic balance training	Balance, control of the body movement	Body posture exercises (forward, backward and sideward), limits of stability, shifting weight from one foot to another, sit-to-stand, movements, and dual-task exercise Four games to move the avatar in the game (couple leaning and avatar motion)	Dynamic balance training	Balance	Controlling body posture in the forward, backward and sideward directions, exploring limits of stability, shifting weight from one foot to another, Sit-to-stand, movements, and included dual-task exercise
	Functional task training	Standing balance	The patients participated in exercises including a namely taking a step and performing and performed sit-to-stand movement	Conventional training following the Dutch Guideline for Parkinson disease	Standing balance	Standing on one leg or with eyes closed, stepping exercises, dual-task exercises, sit-to-stand exercises on the balancing beam or other challenging support surfaces

All studies, excluding (Wall et al. 2015) used a control group. During one-hour sessions, the patients played multiple commercial games from Nintendo Wii Fit Plus Aerobic exercises (i.e. Basic Run) and Balance games (i.e. Penguin plunge, Segway Circuit, island Cycling, rolling down the river, tightrope walk, obstacle course, ski jump, ski slalom and tilt table).

The six studies used a combination of the following measurement approaches:

- Force platform: explained in 3.1.
- Functional Reach Test (FRT): is a single item test developed for balance problems for older adults. In this test, the patients stand upright and are then instructed to reach forward along a yardstick without moving their feet. The maximum distance is then measured by the physiotherapist. A patient with a score of six or less is considered to have a significant increased risk for falling (Duncan et al. 1990).
- Sensory Organization Test (SOT): is a form of computerized dynamic posturography designed to make a quantitative assessment of sensory integrative ability among the three main sensory systems (Szturm et al. 2011). During this test, the patient stands on a dual-force platform plate in a three-sided surround, with the anterior–posterior being recorded. The SOT test is usually made in three independent sensory conditions (i.e. on firm surface, with a sway referenced visual surround and with a sway referenced support surface) with eyes opened and eyes closed (Szturm et al. 2011; Yen et al. 2011). The outcome of the SOT is the equilibrium score (i.e. the average of the centre of gravity for each try), the composite equilibrium (i.e. a weighted average of the six conditions), the sensory analysis ratio (i.e. the computed averages to identify impairments of individual sensory systems), the centre of gravity, and the strategy analysis (i.e. hip and ankles strategy analysis) (Szturm et al. 2011).
- Verbal Reaction Time (VRT): is the delay between the time when the patient sees the instructions and verbally explaining what they are doing (Yen et al. 2011).
- BBS: explained in 3.1.
- Time Up and Go Test (TUG): explained in 3.1.
- Gait Rite Platform: explained in 3.1.
- Activities-Specific Balance Confidence Scale (ABC): explained in 3.1.
- Romberg Test: is a tool to diagnose sensory ataxia, and gait disturbance (Khasnis and Gokula 2003). For this test, the patient must stand upright with their eyes closed. A loss of balance (LOB) is interpreted as a positive Romberg sign (Khasnis and Gokula 2003).
- 10 Meter Walk Test (10MWT): is a test where the patient is asked to walk a short distance (between six and twelve meters) after which their speed result is compared against a table or normative value (average walking speed for men and women by age class) (Chan and Pin 2019).
- Six minutes’ walk test (6MWT): is a test measuring the maximum walking distant of the patient in six minutes (Enright 2003).
- WISCI II: is a tool to measure improvements in walking ability for spinal cord injury. The therapist observes the patient walking and gives a score out of 20 (Dittuno and Dittuno 2001).
- Rand 36-Items Short Form Survey (RAND SF-36): is a self-reporting questionnaire composed of a set of generic, coherent, and easy quality of life measurements (Hays and Morales 2009).
- Single Leg Stance test: In this test, the patient must stand unassisted on one leg, timed from the time the other foot leaves the ground until the moment the foot touches the ground again or the arms leave the hip (Omaña et al. 2021). If the time result is less than five seconds, the patient has a greater risk of injury from fall (Omaña et al. 2021).
- FES: explained in 3.1.
- Hoehn and Yahr scale: is a scale to evaluate the functional disability associated with Parkinson's disease (i.e. describing the disease through its various stages) and to highlight the severity of the case. The resulting stages are between one (unilateral involvement with minimal or no functional disability) and five (confinement to bed or wheelchair unless aided) (Goetz et al. 2004).
- Unified Parkinson Disease Rating Scale (UPDRS): is a rating tool to gauge the severity and progression of Parkinson's disease in patients. It is separated in four parts: intellectual function, mood and behaviour, activity of daily living and motor examination & motor complication (Hauser et al. 2012; Perlmutter 2009). The UPDRS result can be between one and four with a higher score showing increased severity of the disease (Perlmutter 2009).
- Parkinson’s Disease Questionnaire (PDQ39): is a 39-item questionnaire where a patient self-reports their health status and quality of life (Hagell and Nygren 2007). Essentially, this test assesses how often people with Parkinson's disease experience difficulties across eight dimensions of daily living (including relationships, social situations, and communication etc.). The lower the score, the better the quality of life (Hagell and Nygren 2007)
- Hospital Anxiety and Depression (HAD) scale: is a self-assessment of depression and anxiety (Zigmond and Snaith 1983; Roberts et al. 2014). A score between 0 and 7 is considered normal; a score of 8–10: is considered borderline abnormal (borderline case) while 11–21 is regarded as being abnormal (case) (Zigmond and Snaith 1983).
- Multidimensional Fatigue Inventory (MFI): is a 20-item scale to evaluate five dimensions of fatigue (i.e., general fatigue, physical fatigue, reduced motivation, reduced activity, and mental fatigue) with a total fatigue score between 20 and 100. A high score indicates a high level of fatigue for the patient (Dencker et al. 2015; Shahid et al. 2012).

An analysis of the studies’ results showed that although the FRT score improved in (Van den Heuvel et al. 2014; Wall et al. 2015; Lee et al. 2015) for both the experimental and the control group, there was a greater improvement for the experimental group. Cho et al. (2014) obtained significant balance improvement for both groups and greater overall improvements for the experimental group. Wall et al. (2015) declared no change in the TUG score after the training. This could be explained by the choice of exercises used during the training. The TUG test measures mobility, a complex task requiring many skills, however the Nintendo Wii fit alone doesn’t train many of the mobility skills. Wall et al. (2015) concluded that a lack of activity specificity (i.e. turn, sit-to-stand) within the training was one of the barriers to improving mobility, and by association, the patients’ TUG score. Yen et al. (2011) concluded that the experimental and conventional balance training group obtained better results than the control group (without training) under SOT-5 (unreliable somatosensation with eyes closed). Additionally, the experimental training group (with the technology) significantly improved under SOT-6 (unreliable vision and somatosensation) compared to the conventional training group. The improvement in equilibrium under one somatosensory condition for the experimental and the conventional balance training groups suggests that both groups achieved similar results. Moreover, the non-significant difference between these two groups in the SOT analysis can be related to their similar treatment principles (monitoring visual, somatosensory, and vestibular information during training), a short training duration and a small sample size. In addition, the conventional balance training group also had increases in the vestibular sensory ratio after the training, with no significant effect being recorded for the experimental training.

While the gait analysis performed by Wall et al. (2015) and Van den Heuvel et al. (2014) showed a significant increase in speed, there was no significant correlation between the intervention and its duration on gait and speed. Wall et al. (2015) did not find any significant correlation between and within the groups on the average walking speed and on spatio-temporal gait parameters. Table 6 shows the results of these interventions.

**Table 6:** Summary of measurements and results for Group 2 (Balance board)

Study	Measurement Approach	What was measured	Results
Lee et al. (2015)	Force Platform	Balance	Centre of pressure path length and velocity in the context of EOWB (eyes open wide base) and ECWB (eyes closed wide base) improved in both groups
			The centre of pressure path length and velocity in the EONB (eyes open narrow base) and ECNB eyes closed narrow base) improved in both groups
	FRT	Balance, fall	Score significantly improved in both groups
Yen et al. (2011)	SOT	Balance	No significant difference in the baseline among the groups
			No significant difference between the experimental group and the conventional therapy group
			Better improvement of the equilibrium score of SOT-6 after training and at follow up for the experimental group compared to the conventional therapy group
			Better improvement of the equilibrium score of SOT-5 after training and at follow up for the conventional therapy group compared to the control group
			Significant group x (by) time interaction in condition SOT-5, SOT-6 for the conventional therapy group
			No significant change at SOT-5 and SOT-6 for the control group
			The experimental and conventional therapy groups improved in only one SOT condition (5 and 6); no change was noticed for the control group
			The conventional therapy group increased the vestibular sensory ratio after training
			The conventional therapy group had greater improvments overall compared to the control group
			No significant difference between single and dual task for all three groups was noticed
	VRT	Reaction time, focus	No significant group x (by) time interaction for a single task nor main effect of group and time
			No significant difference for SOT-1
			No significant difference between SOT-1 and SOT-6 in any group
Szturm et al. (2011﻿)	BBS	Balance	Significant improvements within and between groups
			Significant improvement of BBS score for the experimental group
	TUG	Balance, fall	Significant difference between groups, greater completion time for the experimental group
			Improvements in both groups
	LOB	Balance	No significant improvement but significant reduction in LOB counts only for the experimental group
	Gait Rite Platform	Gait	No significant within or between group effect on average walking speed, and spatiotemporal gait parameters
	SOT	Balance, sway	No significant result
	ABC	Balance, fall	Significant improvement of ABC score for the experimental group
Cho et al. (2014)	Romberg Test	Balance	Body centre of pressure movement area of the experimental group with eyes open and closed improved after the training. No improvement for the control group
Wall et al. (2015)	10MWT	Gait, speed	No significant intervention effect nor time effect
			Increase of gait speed
	6MWT	Gait, speed	No significant intervention effect nor time effect
			Increase of gait speed
	TUG	Balance	No significant time effect nor change
	FRT (LFRT, FFRT)	Balance, fall	No significant intervention effect nor time effect
			Increase of FRT score
	BBS	Balance	No significant time effects
	WISCI II	Gait, Spinal Cord injury assessment	No significant time effects
	RAND SF-36	Quality of life	No significant result despite the increasing scores
Van den Heuvel et al. (2014)	BBS	Balance	No statistically significant difference in the changed score
			Slightly better score for the experimental group
	FRT	Balance, fall	No significant difference between the groups
			Slightly better score for the experimental group
	Single Leg Stance test	Balance	No statistically significant difference in the changed score
			Slightly better score for the experimental group
	10MWT	Gait, speed	No statistically significant difference in the changed score
	FES	Fall	No statistically significant difference in the changed score
			Slightly better score for the experimental group
	Hoen and Yahr stage	Parkinson assessment	No statistically significant difference in the changed score
			Slightly better score for the experimental group
	UPDRS	Parkinson assessment	No statistically significant difference in the changed score
			Slightly better score for experimental group
	PDQ39	Parkinson assessment	No statistically significant difference in change score
			Slightly better score for the experimental group
	HAD	Quality of life	No statistically significant difference in the changed score
			Slightly better score for the experimental group
	MFI	Quality of life	No statistically significant difference in change score
			Slightly better score for the experimental group

In order of appearance: FRT: Functional Reach Test; SOT: Sensory Organization Test; VRT: Verbal Reaction Time; BBS: Berg Balance Scale; TUG: Time Up and Go; LOB: Loss of balance; ABC: Activity-Specific Balance Confidence Scale; 10MWT: 10 Meter Walk Test; 6MWT: 6 Minute Walk Test; LFRT: Lateral Functional Reach Test; FFRT: Frontal Functional Reach Test; WISCI II: Walking index for spinal cord injury stage 2; RAND SF36: RAND 36-Items short form survey; FES: Fall Efficacy Scale; UPDRS: Unified Parkinson Disease rating scale; PDQ39: Parkinson Disease Questionnaire 39; HAD: Hospital anxiety and depression; MFI: Multidimensional fatigue inventory

The Verbal Reaction Time (VRT) was analysed by Yen et al. (2011) but no significant result was found. The VRT was not reduced during postural control, which can be explained by the fact that the maximum resource capacity of a patient (i.e. the time when their brain is getting overloaded with information) was not reached during the training.

The improvement in RAND-SF36 results for Wall et al. (2015) was not significant. The explanation is that while RAND-SF36 addresses overall health, it may not capture minor changes in mobility.

Five of the studies determined that the experimental training was useful, feasible and positive for the patients (Yen et al. 2011; Szturm et al. 2011; Cho et al. 2014; Wall et al. 2015; Van den Heuvel et al 2014). Yen et al. (2011) concluded that the task-oriented training significantly improved dynamic balance. They also found that both the experimental and conventional balance therapies were useful and effective. This is supported by Cho et al. (2014) with the feasibility of coupled graded dynamic balance exercises on different surfaces with video-game tasks.

Three studies (Cho et al. 2014; Van den Heuvel et al. 2014; and Lee et al. 2015) also concluded that the results from the experimental training did not surpass those from conventional training. Because of the limitations of the studies (i.e. short duration, small sample), it is difficult to generalize. However, the experimental training was not worse than the conventional training because of benefits such as: low cost, accessibility and increase of patient motivation (Szturm et al. 2011; Cho et al. 2014; Wall et al. 2015).

### Group 3. Use of a camera and a screen

Motion detection cameras (i.e. Kinect, IREX, Sony Eye Toy, VRRS and web-camera) provide opportunities for the user to be immersed in the virtual environment unconstrained by a physical device (i.e. no controllers, no glasses to wear, etc.). The Kinect is an RGB-D sensor, initially used as an input device by Microsoft for the Xbox game console (Han et al. 2013). It captures synchronized colour and depth images with an infrared projector and an infrared camera. Studies have shown a satisfactory performance by the Kinect’s skeletal tracking algorithm in posture recognition (Han et al. 2013). However, the detection of body joints is not always completely reliable, especially for cluttered and occluded scenes (Han et al. 2013). The immersive rehabilitation exercise (IREX) system from GestureTek is composed of: a body recognition camera, red gloves, a green screen, a background mat, and a television screen (An and Park 2018). The IREX system places the live real time full body image of the patient onto the screen where they can then see themselves immersed in dynamic virtual reality video games (An and Park 2018). This system is suitable for rehabilitation and balance training (i.e. more natural, and intuitive movement, adaptable to the patients, their needs and abilities, flexibility) (An and Park 2018; Kizony et al. 2005). The Sony Eye Toy is a game-oriented motion tracking sensor for the PlayStation 2 and provides an opportunity to interact with virtual objects (Rand et al. 2008). This system displays the real-time image of the user, so that they can see themselves manipulating virtual objects within a virtual environment superimposed on an image of the actual physical surrounding (Rand et al. 2008). The Virtual Reality Rehabilitation System (VRRS) is a commercial medical device for rehabilitation and tele-rehabilitation (Gianola et al. 2020). Table 7 presents the studies and their population.

**Table 7:** Summary of the population and setup for Group 3 (Camera and screen)

Study	Sample size	Age profile	Care need	Setup
Im et al. (2015)	18	55–80 years old	Motor rehabilitation	Motion capture (Kinect)
An and Park (2018)	10	29–54 years old	Spinal cord injury	Motion capture (IREX)
Khurana et al. (2017)	30	23–40 years old	Post-stroke	Motion capture (Sony PlayStation 2 Eye toy)
Da Gama et al. (2016)	33	20–68 years old	Shoulder abduction	Motion capture (Kinect)
Aung and Al-Junaily (2011)	1	N/A	Motor rehabilitation	Motion capture
Kaharan et al. (2016)	60	18–65 years old	Ankylosing spondylitis	Motion capture (Kinect)
Gianola et al. (2020)	74	45–80 years old	Post TKA	Motion capture (VRRS)
Trombetta et al. (2017)	10	61–75 years old	Post stroke	Motion capture (Kinect)
Andreikanich et al. (2019)	6	36–66 years old	Spinal cord injury	Motion capture (Kinect)
Kizony et al. (2005)	13	21–53 years old	Spinal cord injury	Motion capture (Gesture Xtreme)

Three studies included a mix of young, middle aged and older adults (Gianola et al. 2020; Trombetta et al. 2017; Da Gama et al. 2016). Aung and Al-Jumaily et al. (2011) did not provide information regarding the age of the study’s population. Note: we included three studies which did not reference older adults, (Khurana et al. 2017; An and Park 2018; Kizony et al. 2005). Based on advice from experts in Rheumatology, these studies can be adapted for older patients with osteoporosis. Overall, the ten studies in this group focussed on eight different care needs [i.e. post-stroke rehabilitation (Trombetta et al 2017; Khurana et al. 2017), shoulder abduction (Da Gama et al. 2016), general motor rehabilitation (Aung and Al-Jumaily 2011; Im et al. 2015), orthopaedic rehabilitation post TKA (Gianola et al. 2020), spinal cord injury (An and Park 2018; Andreikanich et al. 2019; Kizony et al. 2005), and ankylosing spondylitis (Kaharan et al. 2016)]. All ten studies required the patient to perform a specific movement to play the game and receive audio and/or visual feedback on their movements. Each study, excluding (Gianola et al. 2020) required a screen and provided real-time feedback to the patients (audio and/or visual).

Seven studies did not mention the existence of a control group (An and Park 2018; Khurana et al. 2017; Aung and Al-Jumaily 2011; Kaharan et al. 2016; Andreikanich et al. 2019; Im et al. 2015; Da Gama et al. 2016). A summary of the interventions for each study is shown in Table 8.

**Table 8:** Summary of intervention for Group 3 (Camera and screen)

#Session/#Week/Duration (min)	Experimental group			Control group		
	Intervention	Focus	Details	Intervention	Focus	Details
Im et al. (2015)						
8–12/ 4/ 30	The patients participate in a series of 3 games designed for the intervention	Balance, range of motion	"*Balloons game*": The patients must touch and burst the balloons with their foot by hip flexion, internal and external hip rotation "*Cave game*": The patients must avoid collisions with stalactites emanating from the ceiling of a cave by flexion and extension of the knees "*Rhythm game*": The patients must touch the virtual plates around them by moving their feet. The plates appear in seven locations: front-left, front, front-right, left, right, back-left, back	No control group		
An and Park (2018)						
18 / 6 / 30	GestureTek training	Balance, range of motion	*Soccer*: Goalkeeper simulation *Conveyor*: Moving virtual boxes from one conveyor to another one at various height *Volleyball*: Beach volleyball simulation *Formula racer*: Racecourse simulation *Airborne*: Parachute landing simulation *Snowboard*: Avoiding obstacles in snowboard down a narrow slope simulation	No control group		
Khurana et al. (2017)						
20 / 4/45	GestureTek training	Body movement, balance, range of motion	*Birds and balls*: Touch balls appearing on the screen with any part of the body to burst them and/or turn them into birds *Soccer*: Soccer goalkeeper Simulation *Snowboard*: Avoiding obstacles in snowboard down a narrow slope simulation	Conventional therapy	Balance, activity of the daily life	The patients participated in activity including typing on a keyboard, (un)tying knot, picking up sticks, catching/throwing balls, taking cloth on/off
Da Gama et al. (2016)						
No information	‘Reaching and catching game dynamic’ training	Shoulder abduction	Catch a virtual ball and put it in the virtual basket next to them	No control group		
Aung and Al-Junaily (2011)						
1/No information /No information	Reaching training	Range of motion	Catch a solid element at the bottom and move it to the right place on the top	No control group		
Kaharan et al. (2016)						
40 /8 /30	Kinect training	Range of motion, balance, functional ability	Kinect adventures, Kinect sports, Kinect sport season 2 (soccer, golfing, volleyball, table tennis)	No training		
Gianola et al. (2020)						
5/ No information /60	VRRS training	Range of motion (knee), proprioception, balance	Knee extension, ball compression (knee flexed), hip abduction on one side, active flexion–extension, hip flexion with knee extended, gluteal bridge exercise, active knee extension, active triple flexion, supine target proprioception (i.e., number draw), active bilateral squat, active hip abduction	Traditional knee rehabilitation	No information	No information
	Knee motion training and functional exercises	Range of motion and Gait	Passive motion system on a Kinetec knee continuous passive motion system, stair negotiation, level walking	Knee motion training and functional exercises	Range of motion and Gait	Passive motion system on a Kinetec knee continuous passive motion system, stair negotiation, level walking
Trombetta et al. (2017)						
No information	Motion Rehab AVE 3D training	Upper limb motor function, Balance, Lower limb, range of motion	*Game 1:* Ball games requiring to perform shoulder abduction and adduction movements, elbow, and wrist extension *Game 2*: catch objects falling from the top of the scene with the feet and perform hip flexion *Game 3*: Ball game requiring to perform shoulder abduction and horizontal adduction, along with elbow and wrist extension *Game 4*: Catch objects with the feet to perform hip abduction and adduction movement *Game 5*: catch objects falling to perform shoulder flexion *Game 6:* Catch objects with knees to perform hip and knee flexion	No control group		
Andreikanich et al. (2019)						
No information	“Tank” game training	Trunk balance	drive/control a vehicle and lean to turn/avoid obstacle	No control group		
Kizony et al. (2005)						
No information	GestureTek training	Balance, range of motion	*Birds and balls*: Touch balls appearing on the screen with any part of the body to burst them and/or turn them into birds *Soccer*: Goalkeeper Simulation *Snowboard*: Avoid obstacles in snowboard down a narrow slope simulation	No control group		

A combination of the following measurement approaches were used:

- BBS: explained in 3.1.
- ABC: explained in 3.1.
- TUG: explained in 3.1.
- Force platform: explained in 3.1.
- FRT: explained in 3.2.
- WISCI-II: explained in 3.2.
- Limit of stability (LOS): challenges patients to move and control their centre of gravity within their base of support. LOS use eight targets (forward, backward, right, left, forward-right, forward-left, backward-right and backward left), randomly highlighted, that the patient must reach by weight shifting (An and Park 2018).
- FRT modified version (used by Kizony et al. 2005): seven scores are measured and calculated for each patient. For FRT1, the patient stands with the right side of their body near a wall. They are asked to raise their right arm to 90° shoulder flexion, with their elbow fully extended, and to lean forward as far as possible. FRT2 is the same exercise but with the left side of the body adjacent to the wall and with the left arm reaching as far as possible. FRT3 is calculated by the sum of FRT1 and FRT2. For FRT4, the patient faces a wall and is asked to reach with their right arm as far as possible to the right. FRT5 is the same as FRT4 but with the left arm to the left. The sixth assessment (FRT6) is in the same position (facing a wall) and the patient is asked to maximally reach with their dominant arm (left or right) to the highest point possible on the wall in front of them. Finally, FRT7 is calculated by the sum of FRT4, FRT5 and FRT6.
- Active knee Range of Motion (ROM): represents how much the knee can bend and straighten on its own (no external force for the active method) (Gianola et al. 2020; Sharma et al. 2021).
- Isometric strength: measures the ability of the patient to hold a sustained contraction against a resistance for a specific duration. It is assessed with a dynamometer (Gianola et al. 2020; Sharma et al. 2021).
- Frequency of medication: measures the need of medication pre-, post-, and during the training (Gianola et al. 2020).
- Visual Analog Scale (VAS): is a pain intensity uni-dimensional Likert-like rating scale (Gianola et al. 2020; Kaharan et al. 2016; Weigl and Forstner 2020).
- Maximum joint angle: represents the distance between the joints represents the maximum angle of the shoulders (Im et al. 2015).
- Number of repetitions: represents how many repetitions the patient can realise without rest (Da Game et al. 2016).
- Response time: is the duration between the moment when the patient is aware of the action and the start of their movement (Kizony et al. 2005; Im et al. 2015).
- Success rate: is the percentage of success corresponding to the number of correct movements during the training (Da Gama et al. 2016; Kizony et al. 2005; Im et al. 2015).
- Pittsburgh rehabilitation participation scale (PRPS): is a one item scale designed to assess a patient's participation in therapy using a six-point Likert scale reflecting the therapist’s observation of patient participation (from 0 (None) to 6 (Excellent)) (Im et al. 2015; Lenze et al. 2004).
- Interview: is carried out to understand the patient’s view of the virtual therapy (fun, motivation, therapeutic value) and their opinion of the system (interface, augmented reality characteristics) (Trombetta et al. 2017; Andreikanich et al. 2019; Kizony et al. 2005; Da Gama et al. 2016).
- Euro-Quality of life five-dimension (EQ-5D): is a standardised measure of health to determine the evolution of patient health and quality of life through five dimensions (mobility, self-care, usual activities, pain/discomfort, anxiety/depression) (Gianola et al. 2020; Rabin and Charro 2009).
- Global Perceived Effect (GPE): is a scale where the patient numerically rates how much their condition has improved or deteriorated since a predefined time point (Kamper et al. 2010).
- Functional independence measure (FIM): is an 18-items instrument to measure a disability (including measures of independence for self-care, locomotion, communication, and social recognition) on seven levels (a higher score indicates greater independence) (Gianola et al. 2020; Kizony et al. 2005).
- Ankylosing spondylitis quality of life (ASQOL): is an 18-items rating scale used to assess the health related quality of life for patients with ankylosing spondylitis. A high score on this scale indicates a poor quality of life (Kaharan et al. 2016; Doward et al. 2007).
- Bath ankylosing spondylitis disease activity index (BASDAI): is a 6-items rating scale from one (no problem) to ten (worst problem) related to fatigue, spinal pain, joint pain/swelling/areas of localized tenderness, morning stiffness duration and stiffness severity (Kaharan et al. 2016).
- Bath ankylosing spondylitis functional index (BASFI): is an instrument to assess the degree of functional limitation in patients with ankylosing spondylitis (Calin et al. 1994; Kaharan et al. 2016)).
- Spinal cord independence measure III (SCIM): is a scale of daily functioning assessment of patients with spinal cord lesion. The SCIM III contains 19 tasks divided in three sub-scales (self-scare, respiration/sphincter management, and mobility) on which the therapist will give a score corresponding to what the patient can achieve (Itzkovitch et al. 2018; Boswell-Ruys et al. 2009).
- T-shirt test: is a time measurement of duration to put on and take off a t-shirt (Khurana et al. 2017).
- Western Ontario and McMaster universities arthritis index (WOMAC): is a self-administered questionnaire consisting of 24 items divided into three sub-scales (pain, stiffness, physical function) and evaluating the hip and knee osteoarthritis. A high score on any of the sub-scales indicates worsening pain, stiffness, and/or functional limitations for the patient (Roos et al. 2009).

Six studies measured the patients’ progress in balance (An and Park 2018; Khurana et al. 2017; Da Gama et al. 2016; Im et al. 2015; Gianola et al. 2020; Kizony et al. 2005). Improvements in the BBS score and the lowest time for the TUG were noticed by An and Park (2018) and Im et al. (2015). The decrease of the TUG time could lead to an increase in standing balance and upright mobility. An and Park (2018) also obtained a significant increase of the overall loss of stability (LOS) score. The forward and backward LOS did not differ significantly, but the directional LOS did. The improvement of the overall and directional LOS means that the adjustment ability improved in the frontal plane due to the weight shifting provided by the training (An and Park 2018).

The patients’ success rate in the game improved for Im et al. (2015), Da Gama et al. (2016) and Kizony et al. (2005), which suggests that the game helped the users to learn the movements and to repeat them correctly (i.e., Da Game et al. 2016). Note:The experimental group usually had 70% more correct results compared to 0% for the control group. The improvement in performance resulted in an increase in the average number of repetitions during the training in the study by Kizony et al. (2005). In the most optimistic scenario, a patient was able to engage in 100 more repetitions while using the system (Da Game et al. 2016). Kizony et al. (2005) used the performance score to conclude that the 'Snowboard' game was the easiest (with the highest score in both groups) while the 'Soccer' game was the most difficult (with the lowest score for both groups but mostly for the patients with spinal cord injury).

A significant increase of the PRPS score to the maximum for every participant was noticed by Im et al. (2015). In four studies (Andreikanich et al. 2019; Trombetta et al. 2017; Kizony et al. 2005; De Gama et al. 2016), the majority of the patients reported interest and an improvement in their motivation for the therapy. However, patients reported feeling some physical and mental discomfort (Kizony et al. 2005) (i.e. fatigue. injury-related pain, embarrassment, lack of realism, eye-hand coordination).

Significant improvements for spinal cord injury of the WISCI II, tee-shirt test (change associated with the group effect) and SCIM III (change associated with time) were observed by Khurana et al. (2017) and An and Park (2018). The WOMAC test used by Gianola et al. (2020) showed a similar pattern between the experimental and control groups. This could be due to the lack of manual treatment in virtual reality.

Five of the studies concluded that virtual rehabilitation was safe, convenient, and more engaging than conventional training (An and Park 2018; Aung and Al-Jumaily 2011; Gianola et al. 2020; Kizony et al. 2005; Da Gama et al. 2016) obtained better outcomes (i.e. balance, fall prevention, spinal cord injury stage assessment) in the experimental group which might be due to the dynamic nature of the moving virtual stimuli. Three studies (Aung and Al-Jumaily 2011; Gianola et al. 2020; Trombetta et al. 2017), considered virtual rehabilitation an efficient, economical, and convenient alternative solution to traditional face-to-face therapy, particularly if the patients accept the technology. Moreover, Gianola et al. (2020) stated that the technology has the advantage of reducing the number of in-person sessions performed in rehabilitation centres. The ability to help in both learning and performing the movements correctly was highlighted by Da Gama et al. (2016) as an important characteristic of an unsupervised situation.

However, improvements can still be made. Da Gama et al. (2016) noted that the entertainment criteria was not consolidated because of the simplicity of the game. In the study of Andreikanich et al. (2019), the doctors and therapists involved in the study proposed some improvements (i.e. progressing skills levels, hand gesture control, adaptability, use of a seat wheel-chaired patients to protect them from falls). Gianola et al. (2020) declared that virtual reality-based training is not superior to tradition rehabilitation in relieving pain and improving other function outcomes. Gianola et al. (2020) concluded that the correlation between VAS and gender could have influenced the result. 60% of the studies reported limitations such as the sample size (An and Park 2018; Kizony et al. 2005), the use of old/cheap technology (Im et al. 2015), the lack of standard exercises and objective muscle power and balance measurement in the follow-up (Karahan et al. 2016) and the use of only healthy patients (Aung and Al-Jumaily 2011).

### Group 4. Other devices

Devices such the CyberTouch Dataglove or the Youkicker rehabilitation system, which focus on a specific part of the body can also be used for exergames. The Youkicker is a VR lower limb rehabilitation system which presents a virtual representation of the feet and the legs in the first-person perspective (Villiger et al. 2017). The CyberTouch dataglove is a tactile feedback instrumented glove (Dimbwadyo-Terrer et al. 2016). Both devices require the use of a screen. Villiger et al. (2017) used the Youkicker to focus on ankle and foot movements whilst Dimbwadyo-Terrer et al. (2016) used the CyberTouch Dataglove and focussed on the movements of the hands and fingers (Table 9).

**Table 9:** Presents the results of each intervention and the feedback from the physiotherapists

Study	Measurement approach	What was measured	Results
Im et al. (2015)	Response time	Performance	Mean response time decreased significantly across the sessions during the balloon game, cave game and rhythm game
	Success Rate	Performance	Increase in the success rate
	Maximum joint angle	Performance	Hip flexion task associated with the highest rate of success
			Average degree of external rotation improved
			Knee flexion angle improved
			Mean knee flexion angle decreased during the rhythm game
	BBS	Balance	Higher score (balance improvement)
	TUG	Balance, fall	Lower time (balance and mobility improvements)
	PRPS	Motivation	Score increased significantly. All patients scored six on the PRPS scale
An and Park (2018)	BBS	Balance	Higher score
	Force Platform	Balance	Overall score significantly increased. Directional score also increased. Forward and backward scores did not differ significantly
	TUG	Balance, fall	Time significantly decreased
	ABC	Balance, fall	Score significantly increased
	WISCI II	Spinal cord injury stage	Score significantly improved
Khurana et al. (2017)	FRT	Balance, fall	Significant change for time and Group x (by) Time
			No significant change for group effect
	Tee-shirt test	Spinal cord injury stage	Significant change for group effect
			No significant change for time and Group x Time
	SCIM III	Spinal cord injury stage	Significant change for time
			No significant change for group effect and Group x Time
Da Gama et al. (2016)	Number of repetitions	Performance	Improvement of the number of times the patient performed the movement during training
	Success rate	Performance	Slight increase but no significant difference in performance
	Interview	User experience, opinion, feelings	All patients appreciated the system
			The entertainment and motivation were enhanced
			The user-interface could have been improved
Aung and Al-Junaily (2011)	EMG sensors data	Body movement	Positive results
Kaharan et al. (2016)	VAS	Pain	Improved for the experimental group but not for the control group
	BASFI	Ankylosing spondylitis stage	Improved for the experimental group but not for the control group
	BASDAI	Ankylosing spondylitis stage	Improved for the experimental group but not for the control group
	ASQoL	Ankylosing spondylitis stage, quality of life	Improved for the experimental group but not for the control group
Gianola et al. (2020)	VAS	Pain	No significant difference in the decrease of the pain score found between the experimental and the control groups
	WOMAC	Pain, stiffness, physical function	Similar pattern between the two groups but the item related to joint rigidity was statistically different in the control group
	Force platform	Balance	Higher score for experimental group
	EQ-5D	Quality of life	No difference between the two groups
	GPE score	Quality of life	
	Frequency of medication	Pain, quality of life	
	Isometric strength	Muscle strength	
	FIM	Quality of life	
	ROM	Muscle strength	
Trombetta et al. (2017)	Interview	Performance	Four patients (out of 10) presented difficulties in terms of spatial orientation; they did not capture the objects
			Four patients had difficulties at the beginning but learned by practicing
			The patients presented a greater ability to complete the task in the third person perspective
			Half of the patients (n = 10) obtained the best performance during the tasks (i.e. they hit everything)
			Four patients got terrible results (i.e. they missed everything)
		Comfort	The patients agreed that the visual and oral feedback was more intuitive helping them understand what to do, whilst also being more immersive
			The patients declared that the TV with the Kinect was more comfortable but less immersive than an HMD
		Interest	Every patient expressed an interest in using technology for older adults
Andreikanich et al. (2019)	Interview	Knowledge and familiarity of the technology	Half of the patients (n = 6) had never used computers, four had never played with computer games; and half was familiar with the concept of virtual reality
		Comfort	All six participants reported that they felt comfortable while playing and all successfully finished the game, enjoyed it, and would play it at home. Some also mentioned that they would prefer to play the game in a two-player competitive mode and/or online
		Doctor and physiotherapist expertise	Doctors and physiotherapists stated that the game as the potential to provide good balance training
Kizony et al. (2005)	Rate of success	Performance	Doctors and physiotherapists proposed improvements in the game such as: progression levels in skills evolution, adding hand gestures to control the avatar, seating the patients in a wheelchair to protect them from falling, and adapting the control of the avatar according to the patient’s condition
			Significantly higher score in Level 1 (birds and balls game)
			No significant difference between Levels 2 and 3 of birds and balls game
			No significant difference between the percentage (%) of success in the birds and ball game and soccer game
			Higher performance in the snowboard game with the lowest score in soccer game
			Significant difference between non-disabled and spinal cord injury group in the birds and ball game and the socce gamer. No significant difference in the snowboard game
	Response time	Performance	Significantly shorter time to complete Level 1 of the birds and balls game
			No significant difference in completion time between Levels 2 and 3 of the birds and balls game
			Significant difference between non-disabled and spinal cord injury group (lower time for spinal cord injury group)
	Interview	User experience, opinion, feelings	Most of the patients enjoyed the experience and felt a high level of presence in the different environments
			The only physical discomfort was fatigue and pain related to the injury, after playing games requiring many movements: soccer game (five patients), birds and ball game (two patients) and snowboard game (two patients). No patient asked to stop playing because of the pain
			Some patients felt discomfort related to the feeling of embarrassment (for one patient), to the lack of realism or unfamiliar feeling (for three patients), to the seated position (for one patient) and the difficulty with the e coordination in the birds and ball game (for one patient)
	FRT	Balance, fall	Some patients were able to reach equally well to both sides of their body, others had a marked asymmetry in their reaching ability
			The patients with a score over the median for FRT3 (total FRT score with the side against a wall) performed better than the patients under the median
	FIM	Quality of life	No difference between the two groups in the rest of the levels

In order of appearance: BBS: Berg Balance Scale; TUG: Time Up and Go; PRPS: Pittsburgh rehabilitation participation scale; ABC: Activity-Specific Balance confidence; WISCI II: Walking index stage 2; FRT: Functional Reach Test; SCIM III: Spinal Cord independence measure part 3; EMG: Electromyography; VAS: Visual Analogue Scale; BASFI: Bath Ankylosing Spondylitis Functional Index; BASDAI: Bath Ankylosing Spondylitis Disease Activity Index; ASQoL: Ankylosing Spondylitis Quality of Life; WOMAC: Western Ontario and McMaster university Arthritis index; EQ-5D: Euro Quality of life five dimensions; GPE: Global perceived effect; FIM: Functional independence measure; ROM: Range Of Motion

The size of the population for the two selected articles varied between nine and 12. The age of the total population ranged between 41 and 74 years. These two articles focussed on only one care need: Spinal Cord Injury (Villiger et al. 2017; Dimbwadyo-Terrer et al. 2016) (Table 10).

**Table 10:** Summary of the population and setup for Group 4 (other devices)

Study	Sample size	Age profile	Care need	Setup
Villiger et al. (2017)	12	41–74 years old	Spinal cord injury	Other device (Youkicker)
Dimbwadyo-Terrer et al. (2016)	9	45–63 years old	Spinal cord injury	Other device (Cybertouch)

A combination of the following measurement approaches was used:

- SCIM III: explained in 3.3.
- WISCI II: explained in 3.2.
- Manual muscle test: explained in 3.4.
- Lower Extremity Motor Score (LEMS): is a scale between zero (complete paralysis) and fifty (normal strength), using the ASIA key muscle in bother lower extremities.
- 10 m walk test (10MWT): explained in 3.2.
- Six minutes’ walk test (6MWT): explained in 3.2.
- Berg Balance Scale (BBS): explained in 3.1.
- Time Up and Go (TUG): explained in 3.1.
- Scale Barthel (BI): is an ordinal scale to measure independence and mobility in ten daily life activities (Mahoney and Barthel 1965) (i.e. bowel control, bladder control, grooming, toilet use, feeding, transfer, mobility, dressing stairs, bathing). The time taken and the physical assistance required to perform each activity are used to assign the value of each item. The higher the score, the greater the patient is able to function independently.
- Patients' Global Impression of Change (PGIC): is a self-reported scale reflecting a patient's belief about the efficacy of a treatment on a 7-item scale (from 'very much improved' to 'very much worse') (Scheman and Ferguson 2009).
- Duration: is a measurement of the time to complete each task to evaluate the speed of the patient and their performance during the training.
- Interview: captures the patients‘ opinion on the training (most challenging games, most attractive games, user experiment).
- Jebsen Taylor Hand Test (JTHFT): is a standardised test composed of seven subsets to evaluate the fine and gross motor hand function (i.e. writing, simulated page turning, lifting small objects, simulated feeding, stacking, and lifting large, lightweight, and heavy object) (Fabbri et al. 2021).
- Nine Hole Peg Test (NPHT): is a test administered by asking a patient to take pegs one by one from a container and then place them into holes on a board as quickly as possible, after which they have to put them back in the container (Wang et al. 2015). The score is based on the time to complete the task (Wang et al. 2015).

A significant improvement in balance and in dorsiflexion was obtained by Villiger et al. (2017). Meanwhile, Dimbwadyo-Terrer et al. (2016) reported improvements in dexterity with a lower time for the experimental group and an increase in time for the control group in the NPHT. This may be explained by the fact that 66% of the patients completed the third session in a shorter time. Hence, the learning of the movements might then have been transferred to real objects. Villiger et al. (2017) also recorded an increase in the patients’ motivation after the training. The patients evaluated the system positively (user-friendliness, visual approach). Patients indicated that they would like to continue to use the system. However, the 10MWT and 6MWT tests showed that the effect on gait was not significant. Some mobility characteristics of the 6MWT were perhaps partially assessed by the TUG, but no significant result was measured in the experimental group. The effect of the experimental training for the spinal cord injury was not significant for either the control or intervention groups (Villiger et al. 2017) while there was a more favourable result for the experimental group in Dimbwadyo-Terrer et al. (2016). This indicates that the dataglove and the exercises were more adapted to the spinal cord condition. The two studies concluded that significant improvements in lower limb, balance and functionality showed the feasibility of the training (Villiger et al. 2017; Dimbwadyo-Terrer et al. 2016). Moreover, despite the fact that the motivation after training was higher, the training programme needed to be more engaging. Dimbwadyo-Terrer et al. (2016) also concluded that it could be possible to have training based on functional reaching movements in a virtual environment (Tables 11, 12).

**Table 11:** Describes the interventions

#session/ #Week /Duration (min)	Experimental group			Control group		
	Intervention	Focus	Details	Intervention	Focus	Details
Villiger et al. (2017)						
16–20/no Information /30–45	Lower limb training	Ankle dorsal flexion, knee extension, leg ad-/abduction, dexterity, motor function	*Footbag*: Football juggling *Hamster splash*: Launch hamsters into a swimming pool *Get to the game-daily leaving activity*: Walk avatar from home to a series of locations, as fast as possible by alternatively lifting one ‘s left and right foot *Star kick*: Knee extension to hit a ball towards a presented star with foot *Planet drive*: Avoid touching the car coming on a roadway towards the feet	No control group		
Dimbwadyo-Terrer et al. (2016)						
**4 /1 /30**	Shoulder movement training	Dexterity, motor function	*Reach and release object 1:* Grip and drop three virtual objects (ball, prism, cylinder) using a virtual hand *Reach and release object 2*: Grip and drop three virtual objects (ball, prism, cylinder) using a virtual hand appearing only when the virtual hand is close to the object *Reach objects*: Reach and touch five objects to make them disappear	Conventional therapy	Upper limb, motor function	Activities of daily life training, upper limb functional exercises, and physiotherapy sessions with assisted-active mobilizations of upper limb and trunk balance exercises
	Conventional therapy	Upper limb, motor function	Activities of daily life training, upper limb functional exercises, and physiotherapy sessions with assisted-active mobilizations of upper limb and trunk balance exercises

**Table 12:** Shows the result from the two studies

Study	Measurement Approach	What was measured	Results
Villiger et al. (2017)	LEMS	Muscle strength	After training: Significant increase
			Four patients reached the minimal clinically important difference (MCID)
			At the follow up: No change observed
	BBS	Balance	After training: Significant increase
			At the follow up: No change observed
	TUG	Balance	After training: Significant increase
			At the follow up: significant increase
	WISCI II	Spinal Cord injury stage	After training: No significant effect
			At the follow up: No change observed
	SCIM III	Spinal Cord injury stage	After training: No significant effect
			At the follow up: No change observed
	10MWT	Speed, gait	One patient was unable to perform it
			After training: No significant effect
			Seven patients met the limits of the minimal clinically important difference (MCID)
			At the follow up: No change observed
	6MWT	Speed, gait	One patient was unable to perform it
			After training: No significant effect
			One patient met the limits of the minimal clinically important difference (MCID)
			At the follow up: No change observed
	PGIC	Quality of life, motivation	Seven patients rated motor function was markedly improved
	Interview	User experience, opinion, feelings	The most attractive games were "Hamster Splash" (45% of the patients), "Star Kick" and Planet drive (18% of the patients each), "Footbag" and "Go to the Game" (9% of the patients each)
			All patients evaluated the system very positively, saying that it was user friendly. They also liked the visuals
			Some patients could imagine using the system for longer than the required four weeks of the study
Dimbwadyo-Terrer et al. (2016)	Muscle Balance	Muscle strength	Clinical change between groups: with a higher score for the experimental group
			The experimental group increased its final score, while the score for the control group decreased
	BI scale	Quality of life	No significant result
	SCIM III	Spinal cord injury stage	Improved results for the experimental and the control groups
	NHPT	Hand function, dexterity	Time score decreased for the experimental group and increased for the control group
	Completion time	Performance	Four patients completed the third session in less time than the first session. They all obtained lower times after training
	JTHFT	Hand function	Clinically significant change in the time to perform the task

In order of appearance: LEMS: Lower Extremity Muscle Score; BBS: Berg Balance Scale; TUG: Time Up and Go; WISCI II: Walking Index Spinal Cord Injury stage 2; SCIM III: Spinal Cord Independence Measure part 3; 10MWT: 10 m walk test; 6MWT: 6 min’ walk test; PGIC: Patients’ Global Impression of Change; BI; Barthel Index; NHPT: Nine-Hole Peg test; JTHFT: Jebson Taylor Hand Test

## Discussion

There is no previous systematic review which explores and analyses existing technology-based approaches and exercises using virtual rehabilitation to support the physical rehabilitation of older adults and post-menopausal women with an emphasis on osteoporosis. Hence, this literature review seeks to identify effective ways to design virtual rehabilitation to obtain physical improvement (e.g. balance and gait) and support engagement (i.e. motivation) for people with skeletal disorders. In our review, we examined: the impact of four types of technology (i.e. HMD, balance board, camera, and more specific devices), the intervention method/s (i.e. exercises), and the measurement approaches with respect to physical improvement (i.e. balance, gait, muscle strength, body movement, dexterity, hand function, care-oriented) and the support of engagement, post-intervention.

Nine studies (Blomqvist et al. 2021; Yoo et al. 2013; Jung et al. 2012; Lee et al. 2017; An and Park 2018; Khurana et al. 2017; Da Gama et al. 2016; Gianola et al. 2020; Villiger et al. 2017) achieved significant overall improvements in balance and fall prevention through the use of an HMD, a body tracking camera or the YouKicker. While overall improvements in balance were also achieved with the balance board, the results were not significant for all studies in this review (i.e. significant results were achieved for Yen et al. (2011) whilst there were insignificant results for Van den Heuvel et al. (2014)), due to the limited mobility of the balance board. Wall et al. (2015) reported that as the balance board did not significantly improve mobility factors (e.g. gait) which are important for balance, there was a resultant lack of improvement in the TUG and other mobility tests. Wall et al. (2015) concluded that due to the limitation of the exercise design, balance improved only in the directional score.

With regard to gait improvement, the experimental groups using an HMD obtained significant improvement, whilst the group using a camera or alternative device (e.g. Youkicker) did not notice a significant effect of the training.

Villiger et al. (2017) noticed that not every patient was able to participate in the 10MWT and 6MWT. Training with an HMD, a Youkicker or a Dataglove appeared to achieve better results in terms of improving muscle strength. Lee et al. (2017) obtained a similar improvement for both experimental and control groups, which means that the improvement came from the exercise and not from the virtual intervention. Specific devices, such as the dataglove and/or Youkicker allowed more detailed work on one part of the body which may explain these results.

Training with an HMD or with a body tracking camera positively impacted overall body movement. Significant improvements in dexterity and hand functioning were found with the use of dataglove in exergames (Dimbwadyo-Terrer et al. 2016). This shows that in targeting specific physical improvements or overall bodily improvements, the choice of device and the precise definition of the training is critical. As an example, the datagloves and the Youkicker only improved a specific part of the body. Meanwhile, a balance board improved some (e.g. postural control, gait) but not all mobility characteristics. While an HMD or a body tracking camera can have a positive impact on full body training, they do not target specific areas. Ultimately, the type of training and its design are more important than the technology in order to have a bigger impact on the patient.

This review also identifies the most ‘effective’ approach to virtual training design in order to achieve physical improvement and support engagement. Effective virtual training design in this context refers to the design of virtual therapy which significantly improves the targeted areas while engaging the patient with their therapy. The user experience and opinion of both the patients and the therapists of the training has been analysed for interventions using HMDs, a camera, or specific devices. Blomqvist et al. (2021) obtained positive feedback on: the duration of the game and the fixed schedule, the entertainment in one of the training programmes, the helpful feedback in the game and the advantages of the technology (e.g. convenient, safe, easy, fun, cost effective and home-based practice capability). Negative feedback was also recorded with respect to training programme design (e.g. difficulty) and technology choice in relation to: device weight, poor fit and difficulty in calibration. Blomqvist et al. (2021) concluded that a fixed schedule may not suit everyone. They also suggested that short, patient adapted exercises can be more applicable to daily life while mitigating against boredom and pain.

The physiotherapists from these studies proposed solutions to avoid user pain and difficulty in games: wearing protective padding between the nose and headset (Blomqvist et al. 2021), providing clear instructions (Blomqvist et al. 2021; Andreikanich et al. 2019) and ensuring that the patients are also able to play the exergames in a seated position (Andreikanich et al. 2019). Universally positive feedback for exergames using cameras was recorded from the context of: entertainment (Aung and Al-Junaily 2011; Andreikanich et al. 2019; Kizony et al. 2005) and the ability to play from home (Kizony et al. 2005).

AR and VR applications for older adults typically target cognitive and physical rehabilitation, treatment of mental diseases etc. As there is a paucity of literature applying virtual rehabilitation to older patients with osteoporosis, our systematic review resulted in the following conclusions for this vulnerable population;

The results presented thematically are:

1. Virtual Therapy

virtual therapy was not only feasible but provided opportunities to improve balance, gait, fall, strength and/or symmetry while increasing engagement for the physical therapy with meaningful exercise.

(2) HDM

Positive feedback provided during the training helps to motivate participants to train more.

(3) Balance board:
(4) Improvement in FRT score, overall balance, no change in the TUG score.
(5) Better improvements in SOT-5 and SOT-6 compared to traditional training.
(6) Significant improvement in Gait analysis.
(7) Body tracking camera:
(8) Improvements in the BBS score and the TUG test.
(9) Significant increase of the overall loss of stability (LOS) score.
(10) A significant increase of the PRPS score to the maximum.
(11) This technology enhanced the participants’ motivation for the therapy.
(12) Overall design
(13) Need for activity specificity (i.e. turn, sit-to-stand) within the training to improve mobility.
(14) Need to include task-oriented training as this significantly improves dynamic balance.
(15) The inclusion of coupled graded dynamic balance exercises on different surfaces with video-game tasks enhances engagement and improves performance.
(16) The use of games embedded within the virtual training supports the users in learning the movements and repeating them correctly.

Therefore, in conclusion, when deciding to use virtual training for older adults with osteoporosis, the main recommendations (based on the above) include:

- The technology choice (i.e. HMD, body tracking camera, specific devices, balance board) must be related to the care need (i.e. improvement required, patient’s needs).
- While balance boards can be used for overall body improvement, they do not significantly improve mobility factors such as gait.
- When using the balance board, it is necessary to design games/exercises which train balance in multiple directions.
- The use of HMDs, a body tracking camera or the YouKicker can support balance improvement.
- HMDs support gait improvement more so than the use of a camera or YouKicker.
- The use of a Dataglove, Youkicker and/or HMD has the potential to significantly improve muscle strength. The Dataglove has the greatest  impact on muscle strength, followed by the Youkicker and an HMD.
- The use of an HMD or body tracking camera can positively impact overall body movement.
- Datagloves can lead to improvements in manual dexterity and hand functioning.
- An HMD or a body tracking camera can result in positive impacts on full body training.
- An HMD or a body tracking camera cannot be used to track specific areas e.g. hand mobility.
- The type of training and its design are more important than the technology selected.
- The training schedule should be personalised to support/enhance engagement.
- Short (i.e. between 15 and 30 min) and frequent (i.e. 2–3 times per week) training programmes can mitigate against boredom and pain.
- When using an HMD, provide protective padding between the nose and headset.
- Cameras, balance boards and specific device are often more comfortable for use during the training programme.
- Provide clear instructions on what is required in the exergames whilst also stating the expected learning outcomes.
- Design the exergames so that the patients can also train in a seated position.
- The design characteristics preferred by patients include: user-friendly interaction, clear instructions and feedback, a comfortable interface and the opportunity for personalization.
- The comfort and immersion of the patient has an impact on the outcome of the training sessions. Note: HMDs are the most immersive technology.

In conclusion, the choice of the technology and the design of the exergames are key items to improving virtual rehabilitation.

## Limitations

Despite the physical improvements wrought by virtual rehabilitation as reported in the 23 studies, there are several limitations which impact generalizability:

- The absence of a control group for nine studies (i.e. (Blomqvist et al. 2021; Yoo et al. 2013; Van den Heuvel et al. 2014; An and Park 2018; Khurana et al. 2017; Aung and Al-Jumaily 2011; Gianola et al. 2020; Andreikanich et al. 2019; Villiger et al. 2017)).
- A small sample size for the experimental group (ranging from 1 to 74) (Aung and Al-Jumaily 2011; Andreikanich et al. 2019; Wall et al. 2015).
- Two studies focussed on healthy patients (Yen et al. 2011; Szturm et al. 2011).
- A broad age range within the groups (from 18 to 85 years).
- The lack of standard exercises in the training (Kaharan et al. 2016).
- The lack of objective muscle power and balance measurements (Gianola et al. 2020).
- The short duration of three studies (Jung et al. 2012).
- The lack of follow up (Da Gama et al. 2016).

A number of the studies were also limited by: their dependence on a hospital setting (Kaharan et al. 2016) (this made it impossible to transfer the training to a home-setting post the study); the double blinded nature of the intervention (Trombetta et al. 2017) (related to the care needs) and the focus of the study on technology cost effectiveness instead of physical improvement (Trombetta et al. 2017).

This systematic review also includes a language bias, as only studies in English or French were reviewed; thus not every study could be included in the systematic review. Additionally, the search on only three databases (PubMed, ACM and Google Scholar) limited the choice of articles included in the study. Moreover, due to the paucity of studies on osteoporosis, experts in Rheumatology (JJC and BW) advised the inclusion of other care needs i.e., Ankylosing spondylitis, spinal cord injury, motor rehabilitation, post-stroke rehabilitation, shoulder abduction. As a result, virtual rehabilitation for these care needs was investigated. Related to this limitation, not every study focussed on older adults due to the wide range of care needs selected, (i.e. some studies related to younger people).

It would also have been interesting to include an analysis of studies regarding cybersickness in this review.

## Conclusion

In this study, a systematic review was conducted to identify effective ways to design virtual rehabilitation to obtain physical improvement (e.g. balance and gait) and support engagement (i.e., motivation) for people with musculoskeletal disorders. A detailed presentation of the technologies has been made, in addition to outlining the training programmes, exergames and respective outcomes achieved.

Four distinct categories of device, enabling different degrees of immersion in a virtual environment for virtual rehabilitation were used in these studies: head-mounted display (HMD), balance board, body recognition camera and more specific devices (e.g. Datagloves, Youkicker). This paper contributes to the body of knowledge on virtual rehabilitation through its set of recommendations on technology choice and exercise training programme design. This knowledge should benefit both clinicians and researchers in the design and implementation of virtual rehabilitation solutions specific to the care needs of patients with musculoskeletal disorders including osteoporosis.

This study enabled us to define a list of recommendations to design virtual rehabilitation for older adults. In summary,

1. The exergames should be user-friendly and provide clear instruction and feedback. Additionally, a personalized experience is usually appreciated by patients and tends to increase their motivation and engagement.
2. The choice of device is dependent on the care need. For global body training, an HMD, camera or balance board is preferred.
3. The comfort and immersion of the patients will have an impact on the results of the training sessions, with HMDs being the most immersive.
4. Training programmes should be frequent but short in duration (e.g. 15–30 min and 2–3 times per week).

This review is important as osteoporosis affects 200 million people worldwide. The use of virtual rehabilitation has been shown to improve a patient’s physical abilities (e.g. balance, gait, muscle strength, body joint) and their motivation. Virtual rehabilitation can be used for patients with osteoporosis or other comparable musculoskeletal disorders with the goal of enhancing rehabilitation. Our review also shows that as there is a paucity of research regarding virtual rehabilitation for older adults with osteoporosis, this is a confirmation that this audience also requires similar HCI considerations.

## Data Availability

All data generated or analysed during this study are included in this published article and its supplementary information files.
